# DEMENTIA STATUS AND PERCEIVED IMPORTANCE OF RELIGIOUS SERVICES OF OLDER ADULTS OVER 11 YEARS

**DOI:** 10.1093/geroni/igae098.2548

**Published:** 2024-12-31

**Authors:** Yufang Tu, Melissa O’Connor

**Affiliations:** North Dakota State University, Fargo, North Dakota, United States; North Dakota State University, Fargo, North Dakota, United States

## Abstract

Religious/spiritual activities positively impact older adults’ health and well-being, and are associated with reduced cognitive decline and dementia progression. The current study explored the connection between dementia status and changes in the perceived importance of religious service participation (RSP) over an 11-year period. We used data from 12 longitudinal waves (2011-2022) of the National Health and Aging Trends Study (NHATS). NHATS is nationally representative of Medicare beneficiaries in the United States. The current study included 41,567 participants at baseline. The outcome variable measured the self-rated perceived importance of RSP on a 3-point scale. Longitudinal multilevel modeling was conducted with dementia diagnosis as a time-invariant predictor (yes/no). Covariates included sex, age group, education level, and self-rated health. The interaction between time and dementia status was also examined. Individuals with dementia had a lower baseline perceived importance of RSP by 0.28 points (estimate = -0.28, p < 0.001) compared to those without dementia. The average trajectory of the perceived importance of RSP significantly declined over time for both individuals without dementia (p < 0.001) and those with dementia (p < 0.001). Notably, individuals with dementia exhibited a faster decline, decreasing by 0.05 points for every 1 unit/year increase, in contrast to 0.01 points (estimate = -0.01) for those without dementia. This study demonstrated that older adults with dementia perceive RSP as less important compared to those without dementia. However, spiritual/religious engagement has been shown to help individuals with dementia cope with their symptoms.

